# Bismacrocycle: Structures and Applications

**DOI:** 10.3390/molecules28166043

**Published:** 2023-08-13

**Authors:** Xu-Lang Chen, Si-Qian Yu, Xiao-Huan Huang, Han-Yuan Gong

**Affiliations:** 1 Hubei Key Laboratory of Pollutant Analysis and Reuse Technology, College of Chemistry and Chemical Engineering, Hubei Normal University, Huangshi 435002, China; yusq826@163.com (S.-Q.Y.); xhuang@hbnu.edu.cn (X.-H.H.); 2 College of Chemistry, Beijing Normal University, Beijing 100875, China

**Keywords:** bismacrocycle, supramolecular chemistry, applications, self-assembly, advanced optical materials

## Abstract

In the past half-century, macrocycles with different structures and functions, have played a critical role in supramolecular chemistry. Two macrocyclic moieties can be linked to form bismacrocycle molecules. Compared with monomacrocycle, the unique structures of bismacrocycles led to their specific recognition and assembly properties, also a wide range of applications, including molecular recognition, supramolecular self-assembly, advanced optical material construction, etc. In this review, we focus on the structure of bismacrocycle and their applications. Our goal is to summarize and outline the possible future development directions of bismacrocycle research.

## 1. Introduction

**Scheme 1:**
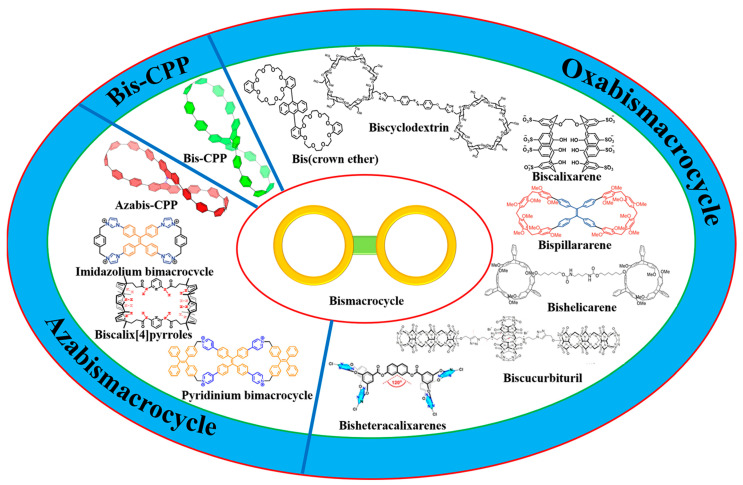
Examples of bismacrocycles and related classification.

In the past six decades, macrocycles with different structures and properties have emerged. These macrocycles include crown ethers [1,2,3], cyclodextrins [4,5,6], calixarenes [7,8], cucurbiturates [9,10], and carbon-rich macrocycles [11,12,13], etc. They have continuously promoted the development of supramolecular chemistry. As important hosts, macrocycles have been widely used in molecular recognition [14,15,16], assembly [17,18,19], molecular machine construction [20,21,22,23], novel material development [24,25,26,27,28], drug exploration [29,30,31], etc. To date, the design and synthesis of macrocycles with special structures and properties is one of the core driving forces of supramolecular chemistry. The combination of multiple cavities and active sites is a promising strategy. Herein, one macrocycle with two cavities is defined as a bismacrocycle. Currently, macrocycle-related studies have been extensive, and their supramolecular chemical properties have also been widely investigated. As the combination result of two macrocycles, a bismacrocycle can effectively expand their properties with high reliability and predictability on the basis of the reports involving its macrocyclic moieties (Scheme 1). However, even some review papers explore related study of specific bismacrocycles [32,33], the summary of bismacrocycles as the subject is lacking [34]. Herein, recent progress of bismacrocycles study is reviewed. It provides an important reference for bismacrocycle-related research (e.g., supramolecular assembly, supramolecular polymer construction, etc.). Specifically, the latest developments in bicyclic compounds since 2017 were summarized for further driving their research development in different fields.

## 2. Oxabismacrocycle

### 2.1. Bis(crown ether)

Smid group synthesized the first bis(crown ether) in 1975 [35], which exhibited stronger interactions with cations (e.g., K^+^, NH_4_^+^) than the corresponding mono(crown ether). It initiated the research on the host with double macrocyclic moieties (i.e., bismacrocycle) in the following decades. To date, a large number of bismacrocycles were generated and widely used in molecular recognition [36] and supramolecular polymer studies [37,38].

**Figure 1:**
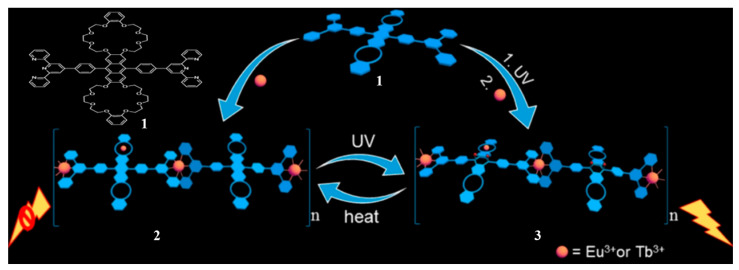
The **1** forms **2** with lanthanide cations, and **1** after UV illumination can further generate **3** with lanthanide cations, and **2** transforms to **3** after UV illumination, and then changes to **2** after heating. Reprinted with permission from ref. [39], copyright 2017 American Chemical Society.

In 2017, Liu group synthesized a bis(crown ether) **1** (Figure 1) [39]. The **1** contained photosensitive 9,10-diphenylanthracene block and terminal pyridinyl groups. It can coordinate with lanthanide metal ions to construct assembly **2**. Under ultraviolet light, **2** can undergo photocatalytic oxidation to form **3** with excellent luminescence performance. Heating induced **3** returning to **2**. The light/thermal regulation between **2** and **3** indicates its good potential to achieve molecular machines and logic gate systems.

**Figure 2:**
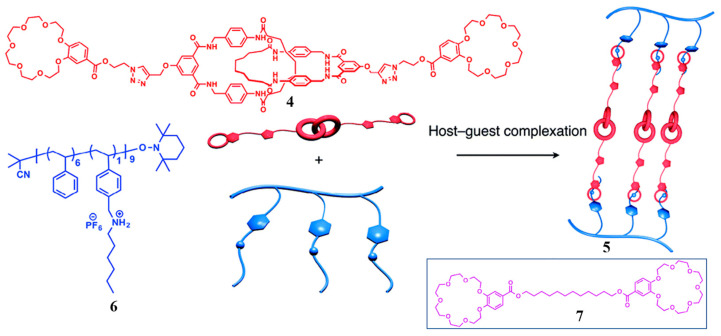
Supramolecular assembly **5** formed between **4** and **6** through host-guest interaction and **7**. Reprinted with permission from ref. [40], copyright 2018 Royal Society of Chemistry.

The novel bis(crown ether) **4** (Figure 2) [40] was synthesized by Xing’s group. As a supramolecular mechanical cross-linker, self-assembly **5** was generated with host-guest interaction between **4** and polymer **6**. It showed significantly higher viscoelastic properties than **7**. It exhibited thermal, pH, and chemical response-modulated gel-sol properties, also outstanding viscoelasticity.

**Figure 3:**
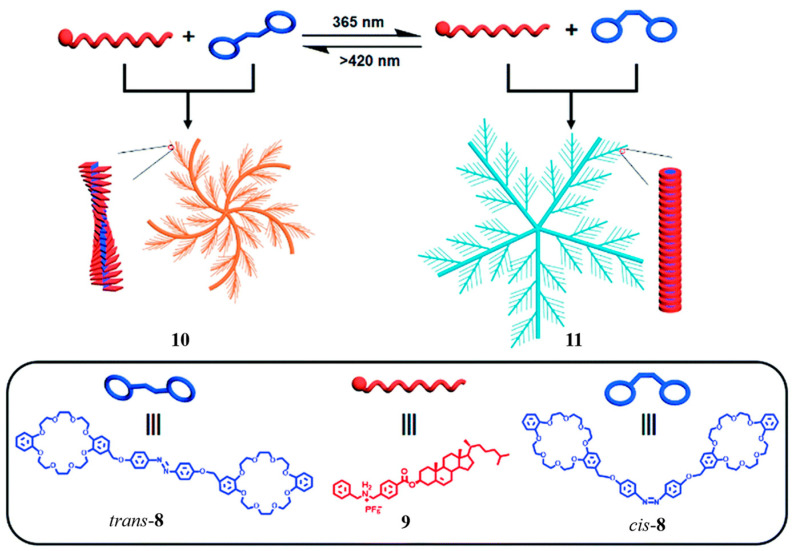
Schematic diagrams of light-controlled interchangeable supramolecular assembly **10** or **11** constructed between *trans*- or *cis*-**8** and **9**. Reprinted with permission from ref. [41], copyright 2019 Royal Society of Chemistry.

In 2019, Liu and colleagues synthesized bis(crown ether) **8** containing azobenzene bridge (Figure 3) [41]. *Trans*-**8** and cholesterol derivative **9** form a snowflake-like supramolecular bell-shaped helix structure **10**. Conversion from *trans*- to *cis*-**8** in **10** under 365 nm UV light created **11**. The **11** has no helical structure and shows concomitant loss of CD signal. Especially, **11** can be transformed as **10** again under illumination (wavelength longer than 420 nm). The morphology and chiral property modulation of supramolecular assembly structures with light can provide en route to subsequent light-driven manual switching and information storage studies.

**Figure 4:**
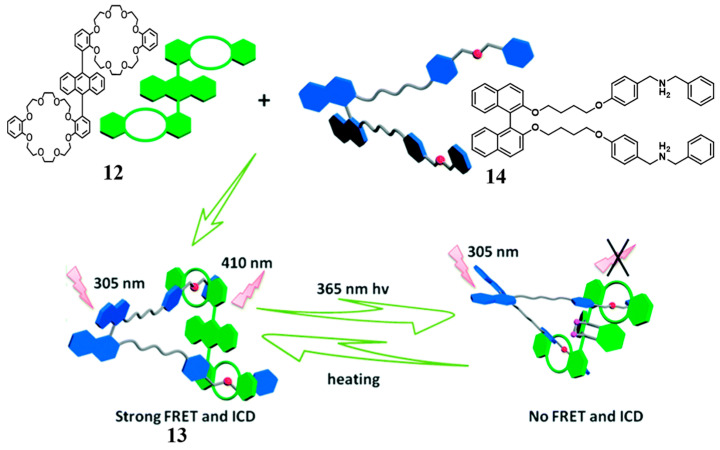
Representation of the stimuli-responsive chirality transfer and FRET between chiral donor **14** and achiral acceptor **12** in **13**. Reprinted with permission from ref. [42], copyright 2019 Royal Society of Chemistry.

Meanwhile, an anthracene-bridged bis(crown ether) **12** (Figure 4) [42] was reported by the Liu group. A novel photochromic pseudo[3]rotaxane **13** was formed between **12** and (R/S)-2,2′-binaphthyl secondary ammonium salt guest (R/S)-**14**. The intrinsic chiral transfer from (R/S)-**14** to **12** is accompanied by fluorescence resonance energy transfer (FRET). Photo-oxidation of anthracene on **12** can further modulate circular dichroism (ICD) and FRET of (R/S)-**13** treated with 365 nm UV light or heating. The multi-stimuli reactive chiral transfer materials can be used to developing chiral functional materials.

**Figure 5:**
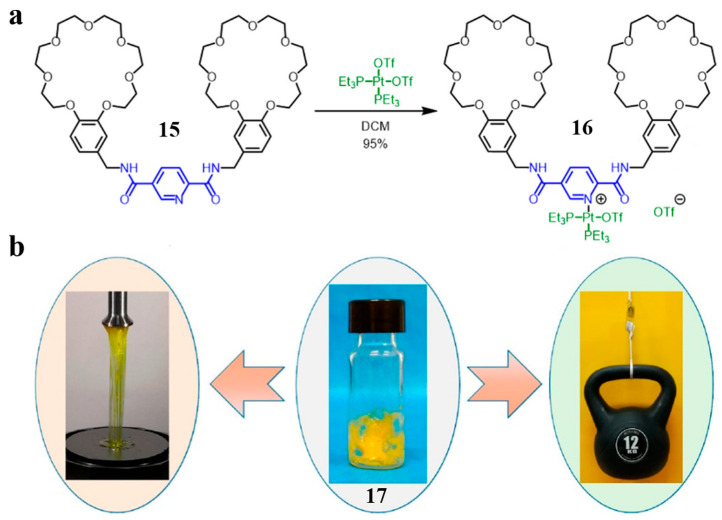
(**a**) Preparation of **16** from **15**; (**b**) the stronger ductility as well as adhesion of **17**. Reprinted with permission from ref. [43], copyright 2019 American Chemical Society.

In the same year, Stang and colleagues synthesized a bis(crown ether) **15** (Figure 5) [43] containing a pyridine linking unit. The **15** and platinum formed the complex **16** (Figure 5a). Hydrogen bonding interactions between **16** and water induced supramolecular polymer **17** with excellent adhesion to hydrophilic surfaces (e.g., glass).

**Figure 6:**
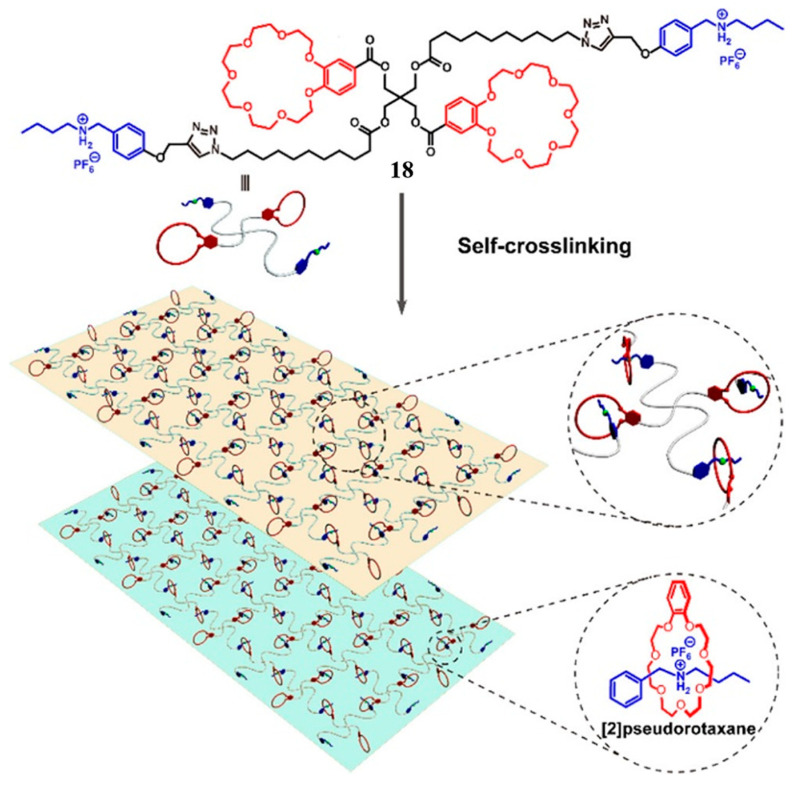
Schematic representation of self-cross-linking supramolecular polymer network formation with **18**. Reprinted with permission from ref. [44], copyright 2020 American Chemical Society.

In 2020, Yan and colleagues obtained bis(crown ether) **18** containing two dialkylammonium salt units. Its self-cross-linking supramolecular polymer network (SPN) formed on the basis of the interaction between its crown ether and dialkylammonium units (Figure 6) [44]. The gel-sol transition of SPN can be modulated with temperature or pH. This study is instructive for developing gel materials with non-covalent interactions.

### 2.2. Biscyclodextrin

Cyclodextrins and their derivatives are widely used in supramolecular self-assembly, supramolecular polymer, and biopharmaceuticals. They are cheap, easily availabile, and non-toxic [4,5,6]. Biscyclodextrins can be prepared via simply linking two cyclodextrin units with linking fragment(s). They can be applied as the building blocks for further self-assemblies [45,46,47,48].

**Figure 7:**
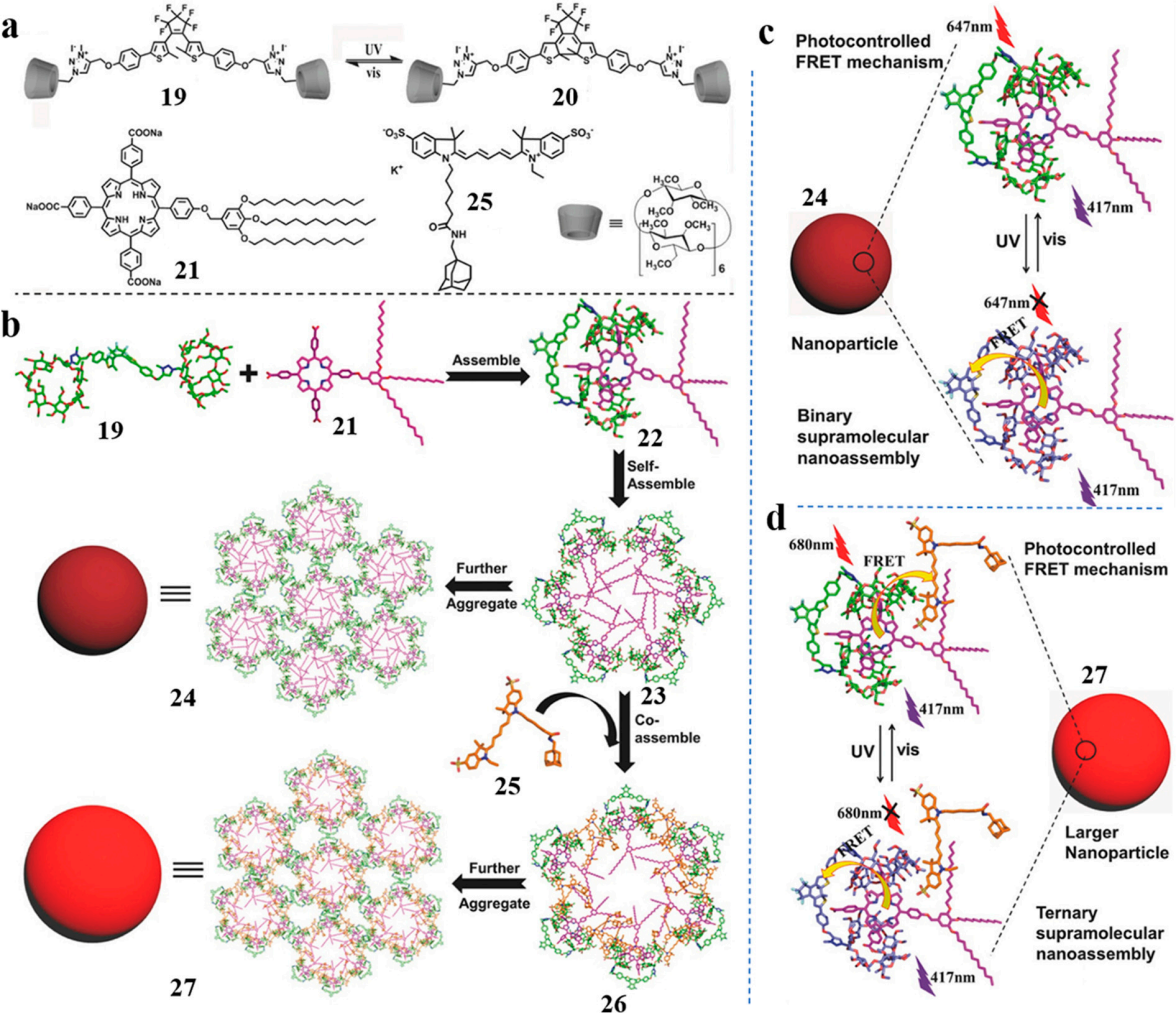
Chemical structure of **19**, **20**, **21**, and **25** (**a**); schematic illustration of spherical nanoparticles (**24**, **27**) formed with **23** and **26** assemblies (**b**), also the photocontrolled energy transfer process in binary assembly **24** (**c**) and ternary assembly **27** (**d**). Reprinted with permission from ref. [49], copyright 2017 Wiley-VCH.

In 2017, Liu group synthesized a biscyclodextrin **19** (Figure 7a) [49] bridged with dithiophene. The **19** is converted to **20** under UV light, and **20** returned to **19** after visible light irradiation. The **19** form **22** with the porphyrin tricarboxylate guest **21**, then **22** further self-assembles to form **23**. The **23** aggregation generated the binary supermolecular nanoassembly **24**. The **23** co-assembles with amphipathic near-infrared (NIR) cyanine fluorochrome **25** to construct **26**, and then further aggregated to obtain a larger ternary supermolecular nanoassembly **27**. The **25** significantly induced **27** fluorescence enhancement (Figure 7b). Nanoparticles **24** or **27** emitted red light at a maximum wavelength at 647 nm or 680 nm under 417 nm excitation, respectively. Both were fluorescence quenched after treating with 254 nm UV light. Subsequently, the luminescence can be restored under >450 nm light (Figure 7c,d). The light-modifiable multivariate nano-assembled structures provides a new strategy to design and develop photoconvertible photoluminescent materials.

**Figure 8:**
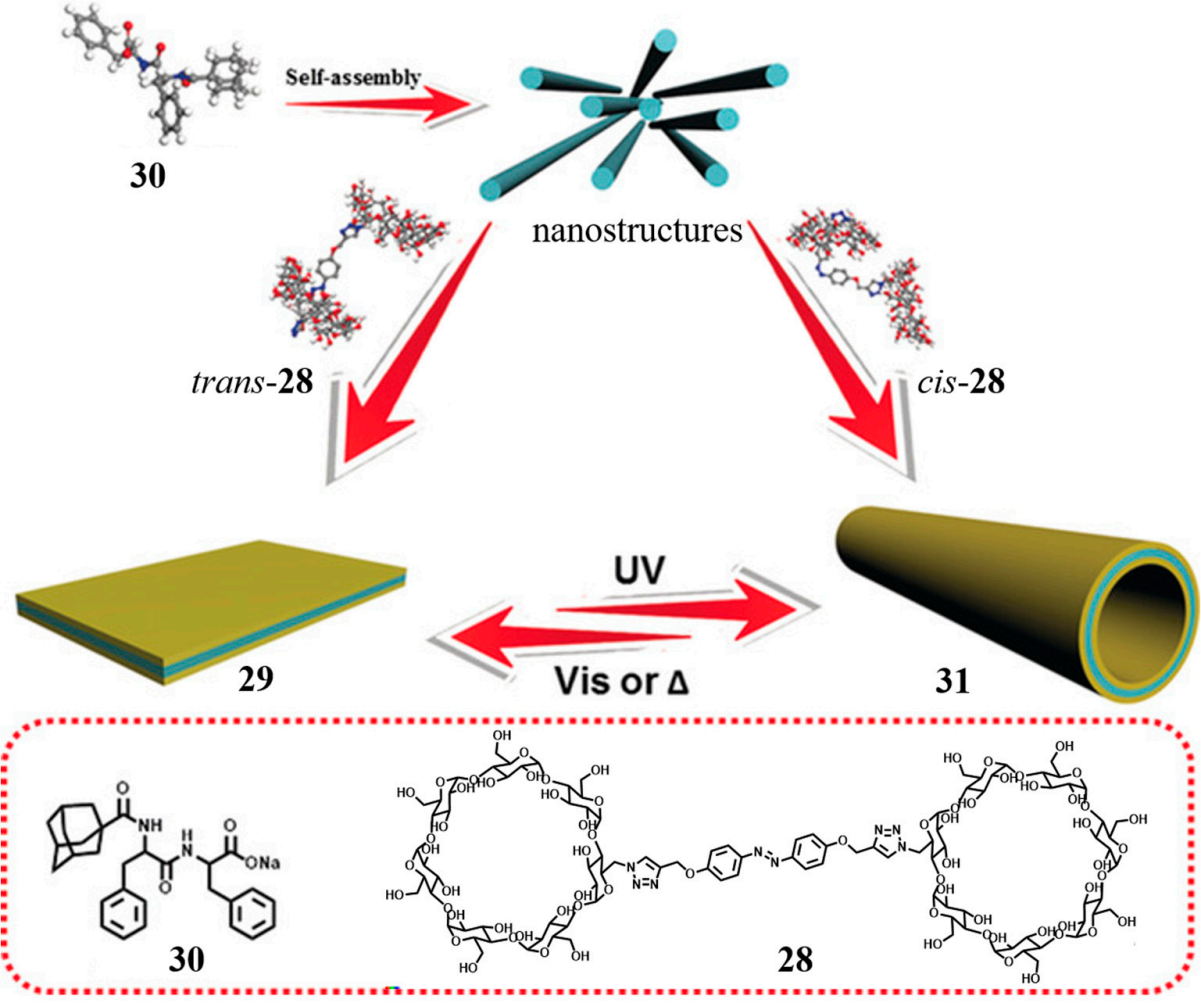
Chemical structure of **28, 30**, and schematic illustration of their assembly. Reprinted with permission from ref. [50], copyright 2017 Wiley-VCH.

The azobenzene unit was used as a bridging unit to generate bis(β-cyclodextrin) **28** (Figure 8) [50]. *Trans*-**28** and adamantanyl-modified diphenylalanine (**30**) form bilayer 2D nanosheets **29**. Differently, *cis*-**28** and **30** forms 1D nanotubes **31**. The **29** and **31** can be converted to each other via UV/visible illumination or heating. This study provides a new way to achieve light control assembly morphology.

**Figure 9:**
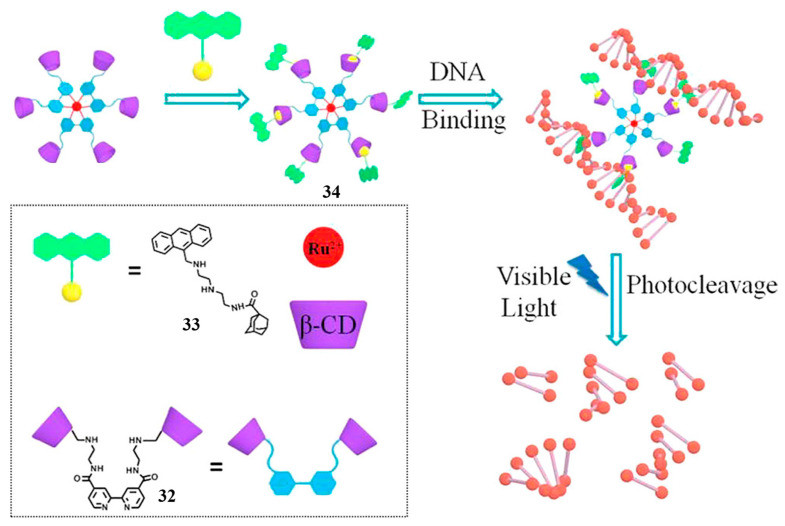
Chemical structure of **32, 33**, and schematic representation of intercalation and photocleavage of DNA using **34**. Reprinted with permission from ref. [51], copyright 2018 American Chemical Society.

The bipyridinyl unit was used as linker to obtain bis(β-cyclodextrin) **32** (Figure 9) [51]. The **32** formed a 3:1 complex with Ru(II), which can bind with adamantane-modified anthracene (**33**) in aqueous solution to form **34**. The **34** can accumulate in the nucleus of cancer cells and induce reactive oxygen species production under visible light irradiation. The net result was effective anticancer activity. This work provided a new strategy for light-driven bright agents for cancer treatment.

### 2.3. Biscalixarene

Calixarene is a class of artificial macrocycles with high application value in chemical, biological, material, environmental, and other multidisciplinary fields due to their low cost, efficient synthesis, and unique properties [7,8]. From the end of the last century, biscalixarenes have been developed for supramolecular assembly and supramolecular polymer construction [52].

**Figure 10:**
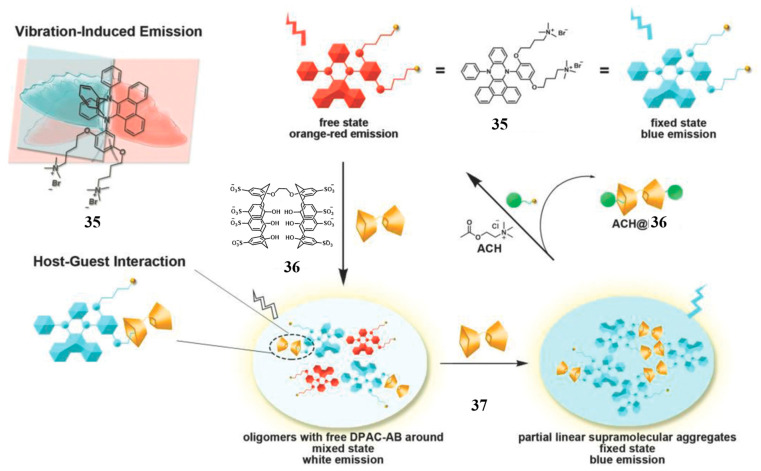
Schematic representation of the preparation from host-guest interactions between **36** and the bifluorescent-emitting VIE guest **35** to generate supramolecular aggregates **37**. Reprinted with permission from ref. [53], copyright 2018 Wiley-VCH.

In 2018, Ma and colleagues reported a novel water-soluble vibration-induced emission (VIE) molecule **35** (Figure 10) [53]. Two quaternary ammonium groups on **35** can insert into the cavity of **36**. Then supramolecular aggregates **37** was created. Free **35** emits orange-red light, while fixed **35** emits blue light. Fluorescence emission from orange-red to white to blue is achieved via controlling the ratio between **36** and **35**. Acetylcholine (ACH) was added in **37** solutions to displace **35** and thus restore orange fluorescence. Such strategies using reversible supramolecular self-assembly to control the emission of VIE molecules provide new ideas to develop tunable luminescent materials.

**Figure 11:**
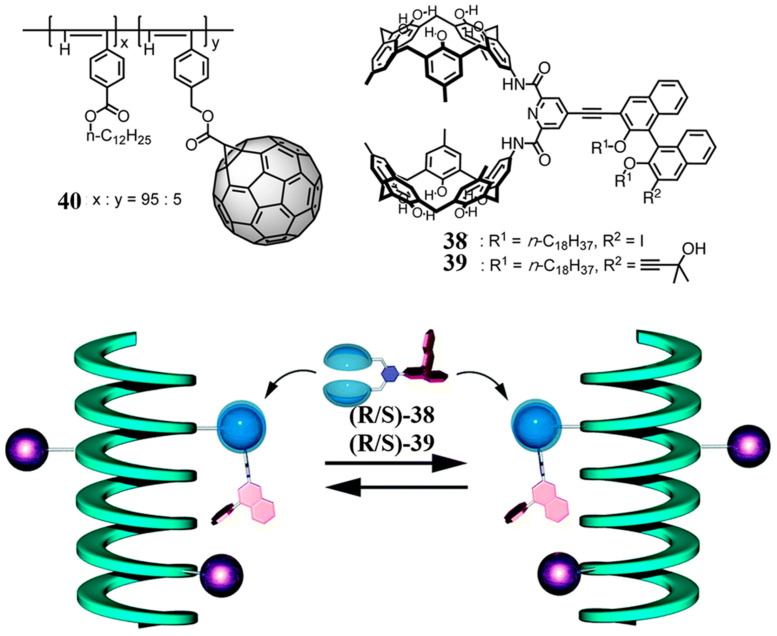
Schematic representation of the helix interconversion of C_60_-appended polyacetylene (**40**) directed by molecular recognition of **38** and **39**. Reprinted with permission from ref. [54], copyright 2020 Royal Society of Chemistry.

**Figure 12:**
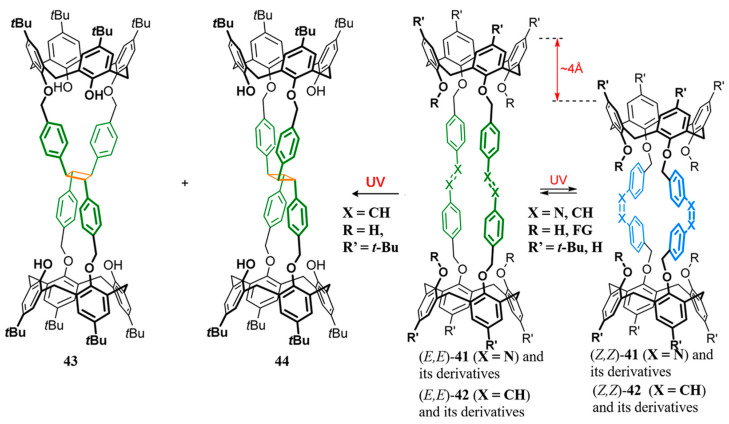
Schematic diagram of the light synthesis of **43** and **44** from (*E,E*)-**42** with the light-regulated length of (*E*,*E*)-**41** and its derivatives. Reprinted with permission from ref. [55], copyright 2023 Royal Society of Chemistry.

Linking two calix[5]arene with chiral binaphthalene provides chiral biscalix[5]arene **38** and **39**. Both interact with C_60_ on C_60_-appended poly-(phenylacetylene) **40** to affect the helix of the polymer (Figure 11) [54]. It is a new approach to control the helical conformation of polyacetylene via host-guest interactions.

(*E*)-azobenzene or (*E*)-stilbene as bridging units link two calix[4]arenes to construct bis(calix[4]arenes) (*E*,*E*)-**41** or (*E*,*E*)-**42** (Figure 12) [55]. (*E*,*E*)-**41** and its derivatives can be converted to (*Z*,*Z*)- forms with a shortened length about 4 Å under 365 nm UV illumination. (*Z*,*Z*)- forms subsequently returned back to (*E*,*E*)-**41** via heating at 50 °C. Tetraester substituted (*E*,*E*)-**42** can be photoisomerized as (*Z*,*Z*)-**42** in the presence of photosensitizer 1,2-benzanthracene. Due to its good stability, it has been separated and cultivated to obtain a single crystal structure. (*E*,*E*)-**42** generates [2 + 2] cycloaddition under 365 nm light in CH_2_Cl_2_ to obtain **43** and **44**. This research provides the material basis to generate complex light-controlled supramolecular assemblies.

### 2.4. Bispillararene

**Figure 13:**
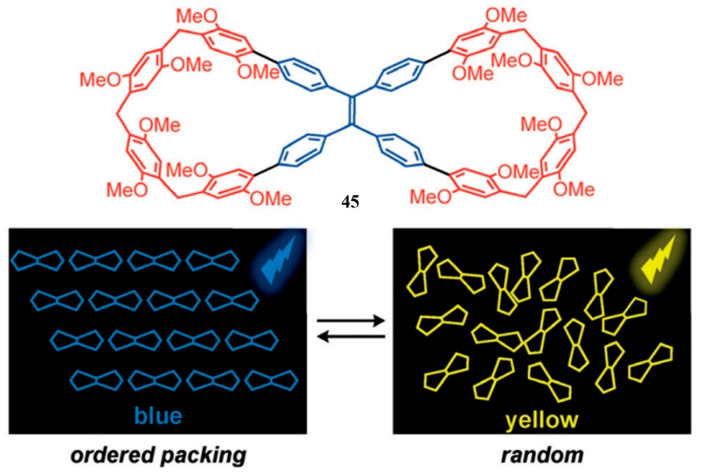
The structure of **45** and its solid-phase fluorescence properties. Reprinted with permission from ref. [56], copyright 2020 Wiley-VCH.

In 2020, Cong group introduced tetraphenylethylene as linker to obtain novel fluorescent bispillararene **45** (Figure 13) [56]. The fluorescence of crystalline **45** changes from light blue to yellow after grinding, and then it can be returned to blue treated with *p*-xylene vapor. Further information encryption patterns can be prepared.

**Figure 14:**
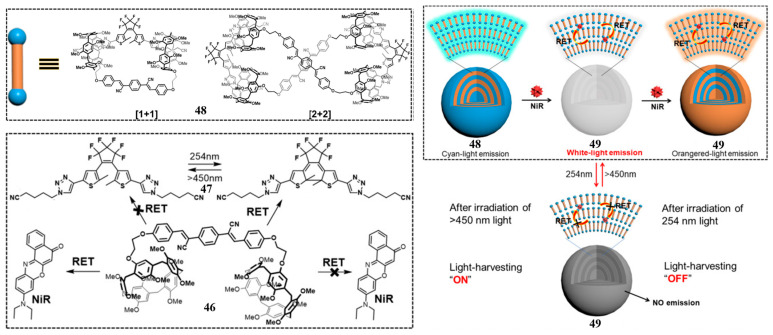
Schematic illustration of the controllable light-harvesting nanosystem based on photo-modulation of the energy transfer pathway. Reprinted with permission from ref. [57], copyright 2021 Elsevier.

In 2021, Liu group reported a novel bispillararene **46** (Figure 14) [57]. The **46** and photosensitive guest **47** form a 1:1 complex **48** with [1 + 1] or [2 + 2] form. Adding Nile red (NiR) to **48** aqueous solutions can obtain three component self-assembled **49**. Increasing NiR ratio gradually changed the fluorescence of **49** from green to white, and then to orange red under 365 nm light. Under 254 nm illumination, the fluorescence of **49** quenched. A total of 450 nm visible light irradiation can restore **49** luminescence (Figure 14). Herein, the reversible morphology of **47** can be controlled via irradiating **49** with different wavelength light. Then the resonance energy transfer (RET) between **46** and NiR can be regulated to modulate the luminescent performance of **49**. This strategy can be further applied to the preparation of photoresponsive supramolecular intelligent materials.

**Figure 15:**
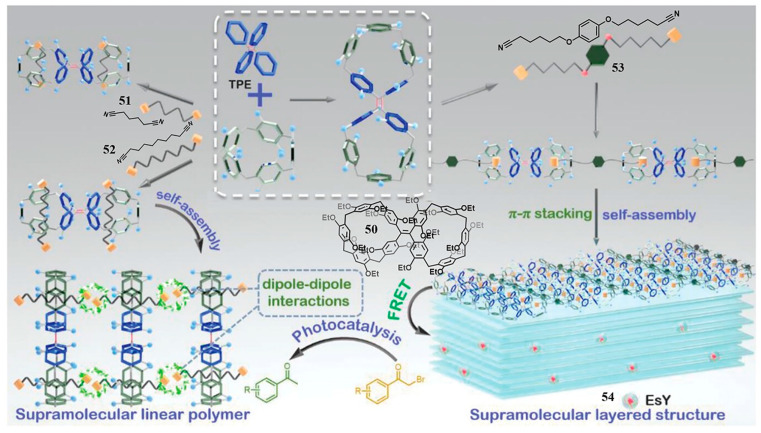
Schematic representation of the controlled assembly and photocatalytic process between **50** and guest **51–53**. Reprinted with permission from ref. [58], copyright 2023 Wiley-VCH.

In 2023, Hu group linked two pillar[5]arenes through C-C double-bonds to obtain bispillar[5]arene **50** containing a tetraphenylethylene (TPE) unit (Figure 15) [58]. Host-guest studies showed that **50** can form a ring-piercing structure with adiponitrile **51** or sebaconitrile **52** in a 1:2 ratio, and then **50** and **52** formed a linear supramolecular polyme. Furthermore, **50** forms a supramolecular layered polymer **54** with **53**, and **54** can be used as a photocatalyst to catalyse the dehalogenation reaction.

### 2.5. Bishelicarene

**Figure 16:**
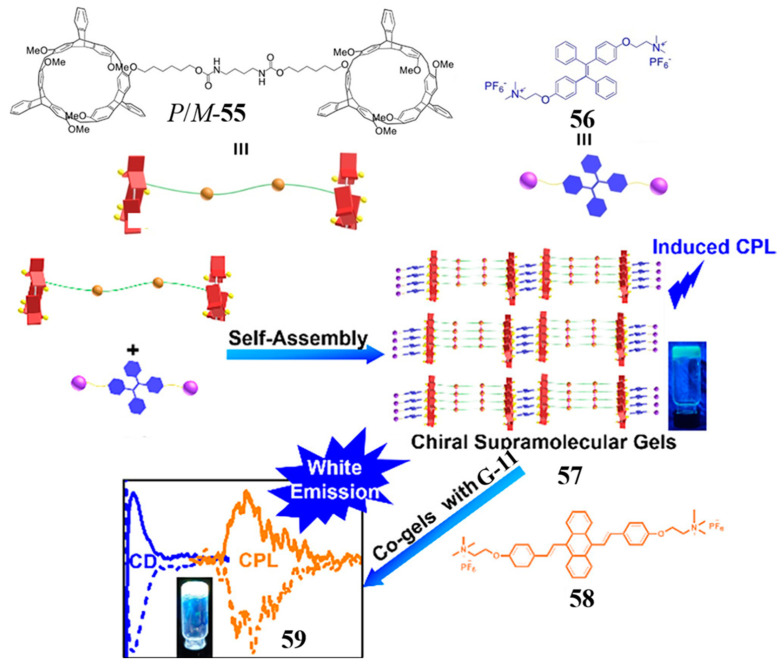
Chemical structure of **55, 56**, and schematic illustration of the supramolecular polymerization of **57** and **59** (inset: photo of the supramolecular gels **57** and **59** under 365 nm UV light). Reprinted with permission from ref. [59], copyright 2022 American Chemical Society.

Chen and colleagues reported a pair of enantiomer bishelic[6]arenes *P*/*M*-**55** (Figure 16) [59] in 2022. It contained two chiral helic[6]arene units. *P*/*M*-**55** self-assembles with guest **56**, a tetraphenylethylene derivative containing two quaternary ammonium units. Finally, a chiral supramolecular polymer **57** formed. The anthracene contained guest **58** combined with **57** to further obtain emission-enhanced supramolecular gel **59**. The circular polarization luminescence (CPL) performance of **59** is induced by the chiral transfer from *P*/*M*-**55** to the non-chiral guest **58**. Furthermore, regulating the ratio of guests **56** and **58** in **59** can emit white light. This work provides a new strategy to construct CPL active supramolecular gel through chiral bismacrocycles.

### 2.6. Biscucurbituril

**Figure 17:**
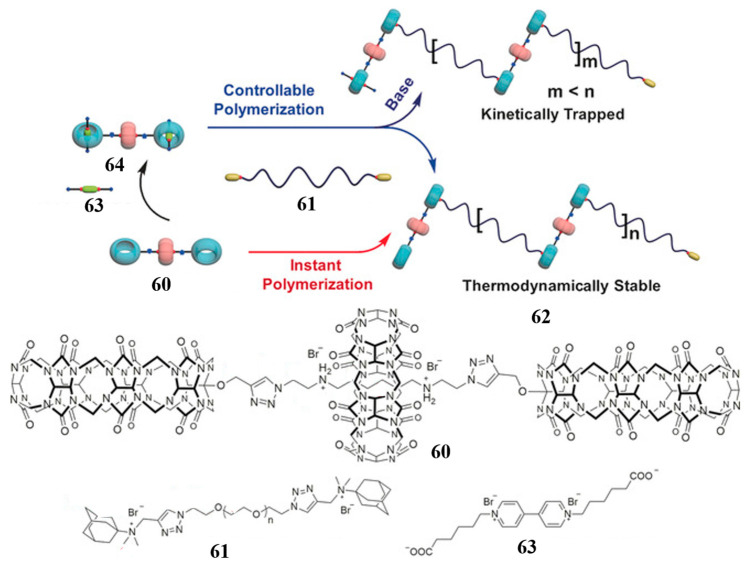
Chemical structure of **60**, **61**, **63**, and schematic representation of the polymerisation degree adjustment between **60** and **61** via the introduction of **63**, a pH-responsive competitive guest. Reprinted with permission from ref. [60], copyright 2017 Wiley-VCH.

Zhang group synthesized the biscucurbit[7]uril **60** in rotaxane in 2017 (Figure 17) [60]. Guest **61** containing N,N-dimethyladamantylammonium (DMAD) on both end groups was added to bind **60**. Supramolecular polymer **62** was rapidly formed with a high polymerization degree. To control the polymerization degree, dicarboxylate guest **63** was used to interact with **60** to form complex **64**. Then **61** was subsequently added in the system to replace **63** due to its stronger affinity with **60**. However, the slow dissociation rate of **63** induced the polymerization degree of **62** can be controlled. When the environment pH was changed, the supramolecular polymerization process was suspended when the pD value increased from 9.7 to 10.8, while the supramolecular polymer remained stable for several days. In this work, pH modulated the dissociation rate of the pre-saturated complex **64**, and further regulated the polymerization degree of **62**.

### 2.7. Bisheteracalixarenes

**Figure 18:**
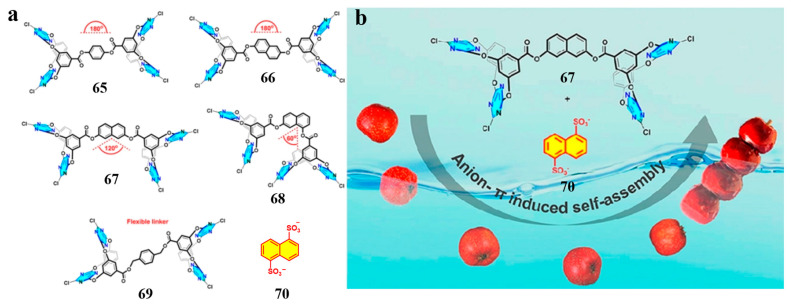
(**a**) Structure of Bisheteracalixarenes (**65–69**) and **70**; (**b**) schematic representation of **67** self-assembling with **70** to form coherent particles via anion-π interactions. Reprinted with permission from ref. [61], copyright 2019 American Chemical Society.

Wang and colleagues used bridging units with different angles and stiffnesses to connect two oxacalix[2]arene[2]triazines to obtain bisheteracalixarenes (**65–69**) (Figure 18a) [61]. The crystal structure of **65–67** and **69** proved that the rigid bridged units can control the orientation of the two large ring cavities, and further modified the stoichiometry of the complexation between each bismacrocycle and chloride aion. Further supramolecular oligomers between **67** and binary naphthalene-1,5-disulfonate anion (**70**) was generated (Figure 18b). The coherent self-assembled particles formed via mixing **67** and 1 molar equiv. of **70**. This study was carried out by rational design of electron-deficient bismacrocycle building units to produce the challenging anion-π interaction-directed self-assembly.

### 2.8. Other Oxabismacrocycle

**Figure 19:**
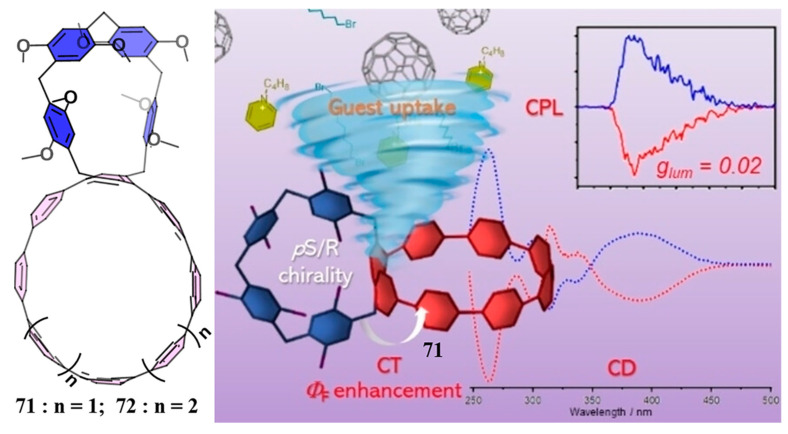
The structure of **71** and its luminescent properties. Reprinted with permission from ref. [62], copyright 2023 Wiley-VCH.

In 2023, Yam group synthesized two multifunctional bis-CPPs **71** and **72** via integrating pillar[5]arene into the [n]CPP backbone precisely (Figure 19) [62]. Compared with [n]CPP, the photoluminescence quantum yield (Φ_F_) values of **71** and **72** substantially increase. Furthermore, **71** shows good circularly polarized luminescence (g_lum_ ≈ 0.02).

## 3. Azabismacrocycle

### 3.1. Pyridinium Bismacrocycle

Pyridinium macrocycles (e.g., “blue box” and its derivatives) have been used for a wide range of applications in supramolecular structures, host, and guest chemistry, catalysis, extraction and sequestration, and molecular electronics due to their unique structures and properties [63]. In addition, pyridinium bismacrocycles have also shown promising applications in supramolecular self-assembly, anion recognition, and bioimaging.

**Figure 20:**
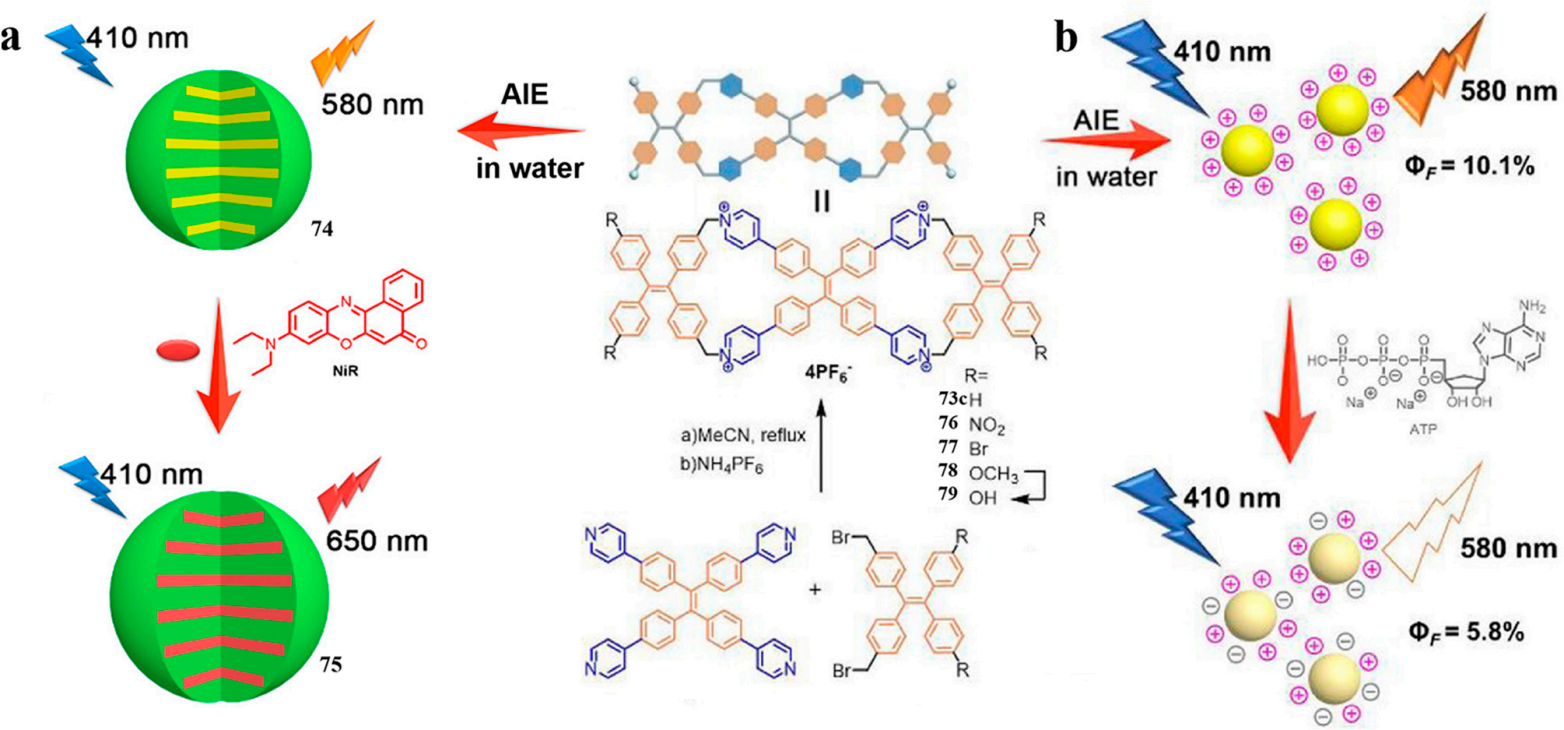
Synthesis of **73, 76–79** and schematic diagram of the preparation of AIE fluorescent nanomaterial **74** and **75** (**a**), schematic diagram of the preparation of AIE fluorescent nanomaterial (**b**). Reprinted with permission from refs. [64,65], copyright 2019 American Chemical Society and 2021 Elsevier.

In 2019, Cao group synthesized the aggregation-induced emission (AIE) pyridinium bismacrocycles **73** containing three tetraphenylethene (TPE) units. (Figure 20a) [64] **73** emits orange light (cantered at 595 nm) in acetonitrile with Φ_F_ as 19.7%, while it emits strong yellow light (cantered at 580 nm) in water with a Φ_F_ as 97.7%. The fluorescence intensity of **73** changed very little with the percentage of water in acetonitrile lower than 80%, and increased rapidly with the percentage above 80%. The **73** forms nanosphere supramolecular assemblies **74** with diameters ranging from 25 to 77 nm in water, with a maximum emission wavelength of 580 nm (excitation wavelength 410 nm). The addition of NiR to the aqueous solution of **74** leads to the formation of further spherical supramolecular assemblies **75** with an increasing diameter as 35 to 83 nm through highly ordered co-assembly. Due to FRET (Φ_ET_ = 77.5%) between **73** and NiR with a high antenna effect (14.3), the maximum emission wavelength of **75** is redshifted to 650 nm (excitation wavelength as 410 nm). This AIE fluorescent nanomaterial has potential applications in cancer cell imaging and diagnosis/photodynamic therapy. In 2021, this group further introduced four different substituents (i.e., NO_2_, Br, OCH_3_, or OH) on **73** (Figure 20b) [65], resulting in bismacrocycle **76–79** with AIE properties. Electron-absorbing groups on **76** or **77** can prohibit the intramolecular PET process between TPE donor and acceptor so that induced enhanced fluorescence. Conversely, **78** and **79** containing electron-donating groups cannot prohibit intramolecular PET process and then cause fluorescence bursts. The **76–79** can also self-assemble into nanospheres in MeCN or H_2_O (1% MeCN). ATP can be encapsulated in the cavities or gaps of the nanospheres formed with **73**, **76–79**. ATP binding leads to a significant fluorescence reduction in **76**, which can be used to detect ATP.

**Figure 21:**
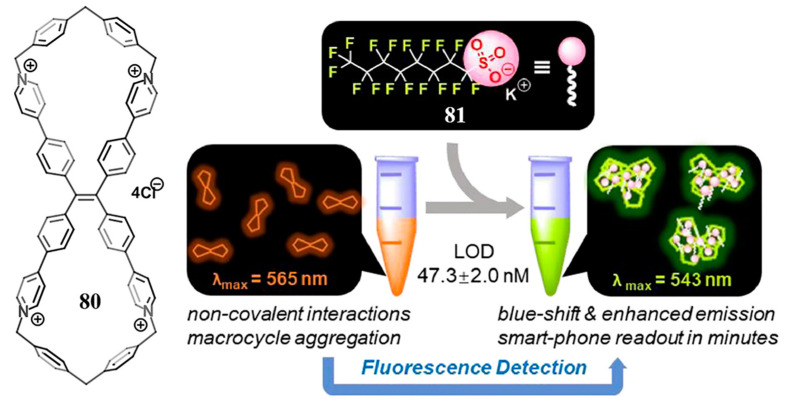
Structure of **80** and schematic representation of **80** as a fluorescent sensor to detect **81**. Reprinted with permission from ref. [66], copyright 2022 Elsevier.

Cong group use a similar strategy to create a water-soluble AIE pyridinium bismacrocycles **80** (Figure 21) [66]. The **80** showed a constant weak yellow fluorescence in aqueous solutions up to 80% THF, while above 90% THF, the fluorescence increased significantly with a blue shift (Δ*λ*_max_ = 46 nm) and the solution became turbid due to the aggregation of **80**. Perfluorooctane sulfonate (**81**) binds **80** in aggregation form following with increasing red shift fluorescence intensity (Δ*λ*_max_ = 22 nm). The **80** exhibits excellent specific recognition of **81** in a variety of anions or cations. This property was further used to visualize and quantify PFOS via smartphone with a detection limit of 47.3 ± 2.0 nmol/L (25.4 ± 1.1 µg/L).

**Figure 22:**
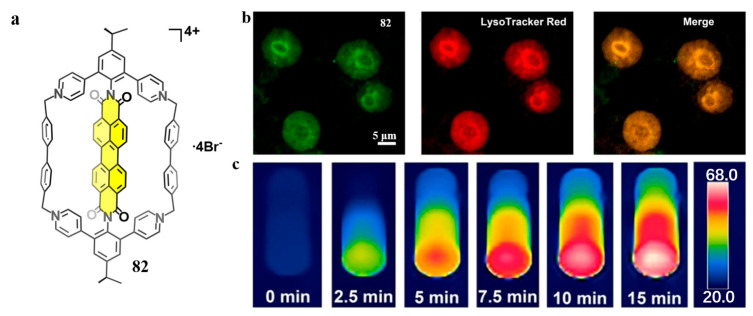
(**a**) Structure of **82**; (**b**) colocalization images of **82** with LysoTracker Red in RAW 264.7 cells; (**c**) photothermal images of **82** (0.1 mM) in the presence of *E. coli* under irradiation at 1 W cm^−2^ for different times. (**b**,**c**) Reprinted with permission from ref. [67], copyright 2022 Wiley-VCH.

In 2022, Zhao and colleagues synthesized **82** (Figure 22a) [67], a water-soluble pyridinium bismacrocycles containing a perylene diimides (PDIs) core. The **82** was encapsulated by double cationic molecular straps on both sides of PDI so that prevents PDI aggregation induced fluorescence quench even at high concentration (e.g., 2 mM) (Figure 22a). Cell fluorescence imaging studies showed that **82** co-localized with the lysosomal labelling dye LysoTracker Red in RAW 264.7 cells with an overlay coefficient as 0.98 and cell viability greater than 95% at all concentration tests (1–200 μM) (Figure 22b). PDI radical species from **82** reduction remained stable at room temperature and heat (60 °C), exhibited photothermal performance without significant decay for 20 cycles under 808 nm radiation. PDI in **82** can be converted in situ to PDI radicals with hydrogenases on the surface of the anaerobic bacterium *E. coli*, and can rise from 25 °C to 68 °C in 15 min under 808 nm laser radiation (Figure 22c). This work provides a new strategy to design and synthesis water-soluble non-aggregated organic dyes.

**Figure 23:**
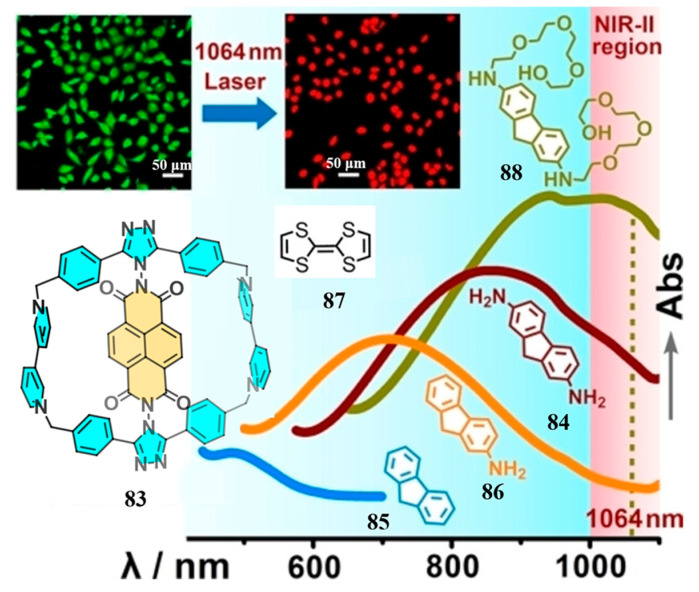
Confocal fluorescence imaging of the photothermal ablation induced by **88**_2_ ⊂ **83** upon irradiation at 1064 nm (**top left**); UV/Vis-NIR spectra of **84**_2_ ⊂ **83**, **85**_2_ ⊂ **83, 86**_2_ ⊂ **83**, and **88**_2_ ⊂ **83**. Reprinted with permission from ref. [68], copyright 2023 Wiley-VCH.

A similar strategy can be applied to design more bismacrocycle. In 2023, Wei and colleagues generated a water-soluble pyridinium bismacrocycles **83** (Figure 23) [68] containing a naphthalene diimide core. The **83** acts as an electron-deficient host that binds strongly to electron-rich guests such as water-insoluble 2,7-diaminofluorene (**84**), fluorene (**85**), 2-aminofluorene (**86**), tetrathiafulvalene (**87**), and water-soluble oligoethylene glycol chain substituted 2,7-diaminofluorene (**88**) in a 1:2 host-guest stoichiometry ratio. The maximum absorption wavelengths of guest_2_ ⊂ **83** were found to be 482, 712, 860, and 1063 nm for the case involving **85**, **86**, **84**, and **87**, respectively. The results suggested that guests with stronger electron donating ability can induce greater charge transfer (CT) absorption redshifts. Upon 1064 nm laser irradiation, aqueous solutions of **84**_2_ ⊂ **83**, **87**_2_ ⊂ **83**, or **88**_2_ ⊂ **83** exhibited significant warming with thermal conversion efficiency value as 37.6%, 39.9%, and 47.4%, respectively. Upon 1064 nm laser irradiation, the non-toxic **88**_2_ ⊂ **83** completely killed HeLa cells, and *E. coli* and *S. aureus*, achieving efficient NIR-II photothermal conversion for cancer cell and bacterial ablation (Figure 20 top left). This study provides new avenues to design and apply biocompatible NIR-II light absorbers with well-defined structures.

**Figure 24:**
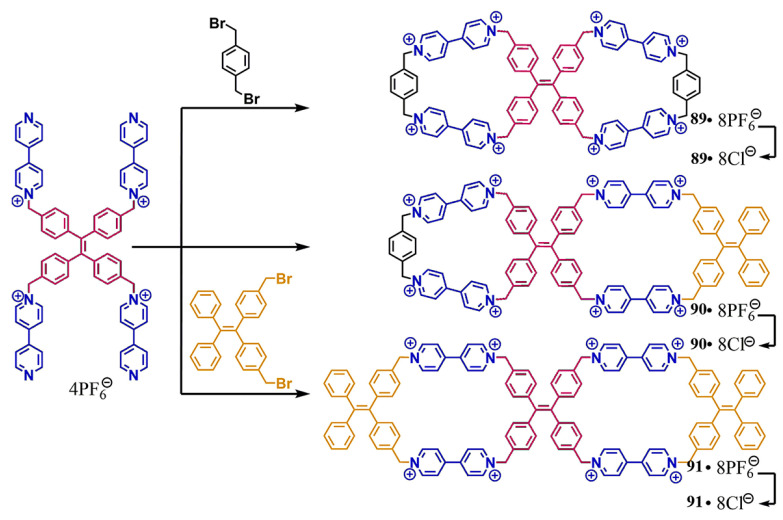
Schematic synthesis of three TPE-containing pyridinium bismacrocycles **89–91**. Reprinted with permission from ref. [69], copyright 2023 Elsevier.

Recently, Cao and colleagues reported three TPE-containing pyridinium bismacrocycles **89–91** via a one-step S_N_2 reaction (Figure 24) [69]. The **89–91** binds nicotinamide adenine dinucleotides (NAD/NADH) in a 1:2 ratio in aqueous solution, with NAD in the open form and NADH in the folded form. This study provides an idea to regulate the conformation of NADH in organisms through host-guest interactions.

### 3.2. Biscalix[4]pyrroles

Calix[4]pyrrole is widely used to bind anions and ion pairs [70]. One or more bridging “walls” connecting two calix[4]pyrrole provide biscalix[4]pyrroles that are widely used in ion recognition, sensing and logic gate structures [33].

**Figure 25:**
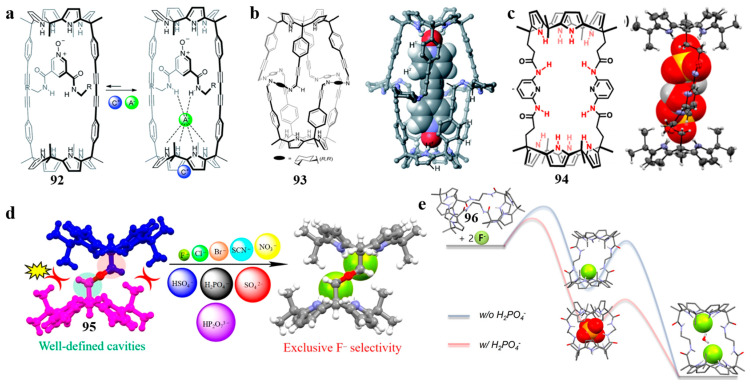
(**a**) Construction of anionic regulated multi-component self-assembly structures based on biscalix[4]pyrrole **92**; (**b**) The structure of **93** and its assembly with double N-oxides; (**c**) The structure of **94** and its assembly structure with two SO_4_^2−^; (**d**) **95** specific recognition of F^−^; (**e**) **96** forms a 1:2 complex with F^−^ through different pathways. (**a**–**e**) Reprinted with permission from ref. [71,72,73,74,75], copyright 2017 Royal Society of Chemistry and 2017, 2020, 2022 American Chemical Society.

In 2017, Ballester group created [2]rotaxane based on biscalix[4]pyrrole **92** and 3,5-bis(amido)pyridine-N-oxide derivatives [71]. Additional anion can modulate the decomposition and combination of multi-component self-assembled structures (Figure 25a). In the same year, this group introduced chiral 1,2-substituted aliphatic diamines to the bridging unit to obtain the chiral biscalix[4]pyrrole **93** (Figure 25b) [72]. The **93** can form supramolecular capsules with double N-oxides with unprecedentedly efficient chiral transfer. Meanwhile, Sessler group generated biscalix[4]pyrrole **94** with a large-cavity (Figure 25c) [73]. The **94** can capture two monovalent H_2_PO_4_^−^, two divalent SO_4_^2−^ and two trivalent HP_2_O_7_^3−^ anions simultaneously. In 2020, He and colleagues synthesized biscalix[4]pyrrole **95** that specifically recognizes F^−^ (Figure 25d) [74]. In 2022, Kim and colleagues synthesized biscalix[4]pyrrole **96** that binds F^−^ and acetate anions in solution with a 1:2 acceptor/anion ratio, and forms 1:1 complexes with oxygenated anions such as C_2_O_4_^2−^, H_2_PO_4_^−^, SO_4_^2−^ and HP_2_O_7_^3−^ (Figure 25e) [75].

### 3.3. Imidazolium Bismacrocycle

**Figure 26:**
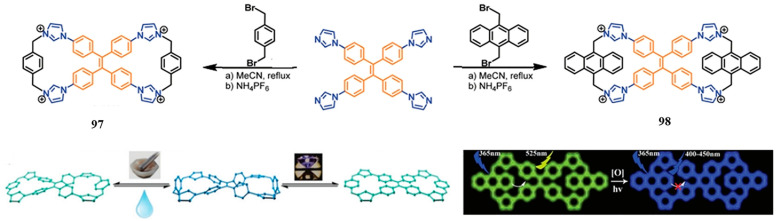
Synthesis of **97–98** and related fluorescence properties. Reprinted with permission from ref. [76], copyright 2020 Royal Society of Chemistry.

In 2020, Cao group synthesized imidazolium bismacrocycles **97** and **98** (Figure 26) [76] with TPE core induced AIE properties. The **97** and **98** show good emission in a wide range of solvents. The **97** changed its emission colour from blue to green (Δ*λ* = 12 nm) after grinding, and returned to blue after fumigation with water vapor. Due to the complete flattening of the benzene ring in the bicyclic structure at high pressure, a large red shift in the emission wavelength (Δ*λ* = ~72 nm) of **97** was observed. Due to the restricted rotation of the anthracene group in **98**, its emission peak does not vary with pressure. An acetonitrile solution of **98** underwent an oxidation reaction under 365 nm light and the fluorescence gradually changed from green to blue within a few minutes. The synthesis of such novel molecules for mechanochromic and photochromic luminescence can provide novel smart luminescent materials.

### 3.4. Azabiscycloparaphenylene

**Figure 27:**
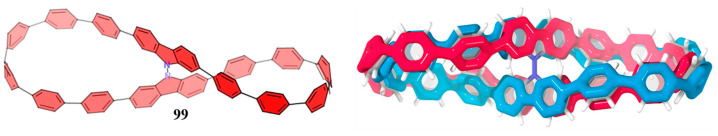
The structure of the **99** and a schematic diagram of its π-electronic system. Reprinted with permission from ref. [77], copyright 2019 American Chemical Society.

Stępień and colleagues obtained the radially conjugated azabiscycloparaphenylene (azabis-CPP) **99** in 2019 (Figure 27) [77]. The 9,9′-bicarbazole core of **99** acts as a stereospecific element giving the entire molecule an “8” twisted structure. The electronic circular dichroism (ECD) spectra of each pure **99** enantiomer contains two major Cotton effects with opposite signs. The curvature control method proposed in this work can reduce the electronic band gap while maintaining a large conjugate length in the nano-hoop system.

**Figure 28:**
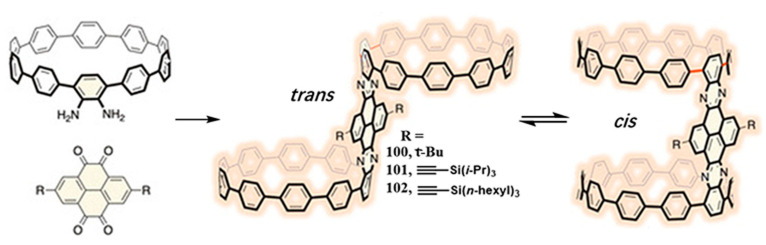
The synthetic strategy of **100–102** and its structure conversion. Reprinted with permission from ref. [78], copyright 2021 Wiley-VCH.

In 2021, Sun group obtained the azabis-CPP **100–102** (Figure 28) [78] with the cyclocondensation between two *o*-diamine-substituted CPP derivative moieties and a 4,5,9,10-tetraketopyrene. The structure of **100–102** rapidly interconverts between *cis-* and *trans*- conformations. Analysis of the NMR hydrogen spectra of the well-solubilized **102** at different temperatures revealed >99% trans structure at temperatures below 183 K. The maximum emission wavelength of **102** in dichloromethane was 616 nm, the brightest fluorophore of the CPP derivatives with *λ*_em_ > 600 nm, and its high quantum yield of 80% was one of the highest values for CPP derivatives. The **102** can bind C_60_ with a 1:2 ratio in solution.

## 4. Biscycloparaphenylene (bis-CPP)

**Figure 29:**
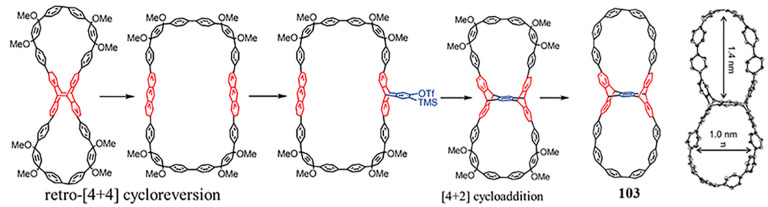
Synthesis of **103** and its single crystal structure. Reprinted with permission from ref. [79], copyright 2019 Wiley-VCH.

In 2019, Cong group synthesized bis-CPP **103** (Figure 29) [79] bridged by benzene rings [80]. The main steps involve the inversion of the dianthracene retro-[4 + 4] cycloreversion, and the ring expansion in the 64-membered macrocycle by transannular aryne [4 + 2] cycloaddition. The crystal structure shows that the two CPPs in **103** linked by the pentiptycene core are ellipsoidal in shape. The **103** precursor was treated with HPLC chiral seperation and further reduction to obtain its enantiomer with an average luminescence dissymmetry factor (glum) as 3.4 × 10^−3^.

**Figure 30:**
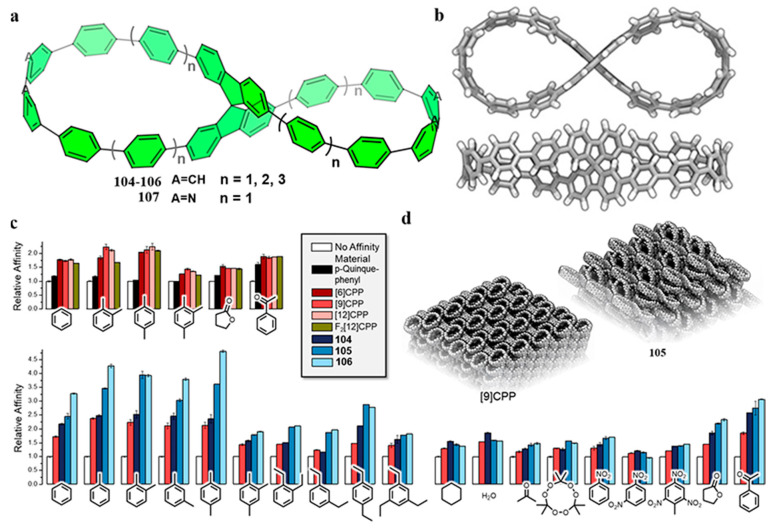
(**a**) The structure of **104–107**; (**b**) The analogue structure of **104**; (**c**) QCM airborne analyte sensing study of the title compounds as affinity materials relative to a passivated surface (denoted as “no affinity material”); (**d**) X-ray crystal structures of [9]CPP and **105**. (**b**–**d**) Reprinted with permission from ref. [81], copyright 2020 American Chemical Society.

In 2020, Jasti and colleagues produced a series of bis-CPPs with 9,9′-spirobifluorene as the core. These bismacrocycles included fluorescent bis-CPPs (**104–106**) with different sizes, and non-luminescent Azabis-CPP (**107**) (Figure 30a) [81]. The porosities of **104–106** were detected as 33.9 m^2^/g, 703.0 m^2^/g, and 11.8 m^2^/g, respectively. X-ray single crystal diffraction experiments showed that **104–105** were loosely supramolecularly arranged, whereas **107** were relatively well-ordered (Figure 30d). Double-cavity CPP had a higher affinity for VOCs (up to 480%) than the single-cavity [9]CPP (Figure 30c,d).

**Figure 31:**
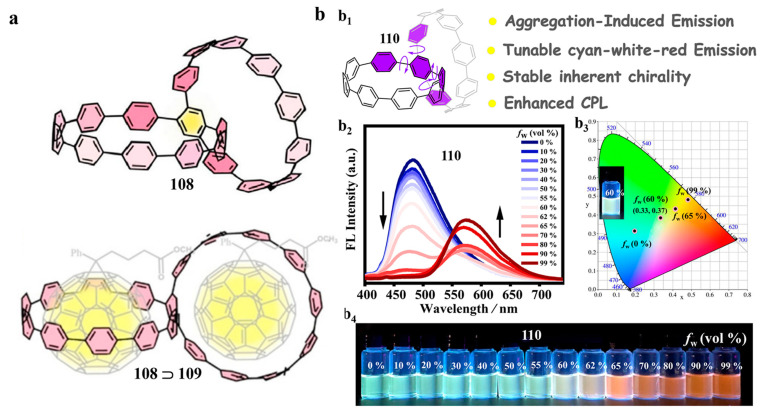
(**a**) The structure of **108** and the peanut-like complexation between **108** and **109** in a 1:2 ratio; (**b**) (**b_1_**) Structure and properties of **110**; (**b_2_**) Fluorescence spectra of **110** in the solvent with different THF/H_2_O ratios; (**b_3_**) CIE 1931 chromaticity diagram of **110** in THF/H_2_O mixtures; (**b_4_**) r Emission colour changes of **110** from cyan to red in aqueous THF with f_w_ = 0–99 vol% under 365 nm UV light. Reprinted with permission from ref. [82,83], copyright 2021 Wiley-VCH and 2022 Nature Publishing Group.

In 2021, Du group generated a highly strained bis-[10]CPP (**108**) (Figure 31a) [82]. The double-cavity twisted structure of **108** was confirmed by scanning tunnelling microscopy. Theoretical calculations show strain energy up to 110.59 kcal mol^−1^, while the central benzene on **108** has a torsion angle as 10.05° and a maximum interphenylene torsion angle as 46.07°. The **108** has maximum absorption wavelength at 352 nm, and maximum emission peak at 523 nm with fluorescence quantum yield as 5% and lifetime as *τ*_1_ = 4.23 ns, *τ*_2_ = 8.46 ns, and *τ*_3_ = 16.93 ns. UV-Vis titration and Job Plot analysis provided the peanut-like binding constants between **108** and **109** as ***K***_1_ = (7.46 ± 0.33) × 10^5^ M^−1^ and *K*_2_ = (5.85 ± 0.25) × 10^4^ M^−1^ with a binding ratio as 1:2. In 2022, Du and colleagues synthesized bis-[8]CPP **110** containing a distorted benzene core similar as **108** (Figure 31(b_1_)) [83]. The **110** fluoresces displayed a maximum emission wavelength at 475 nm under an excitation wavelength as 380 nm in THF, with. The fluorescence quantum yield was ~3% and a fluorescence lifetime was 4.23 ns. Increasing water proportion in THF beyond 60%, **110** showed a new emission band at ~577 nm with gradually decreasing emission at 475 nm (Figure 31(b_2_)). The results indicate that **110** is characterized by both the aggregation-caused quenching (ACQ) and AIE effects, and can induce tunable emission from cyan to red, including near-white light emission (Figure 31(b_3_,b_4_)). The temperature-dependent CD spectra demonstrate the strong stability of both **110** enantiomeric isomers. Moreover, the AIE effect of **110** enhances its CPL properties. This molecule has potential applications as white light emitters, AIE sensors, and chiral luminescent materials.

**Figure 32:**
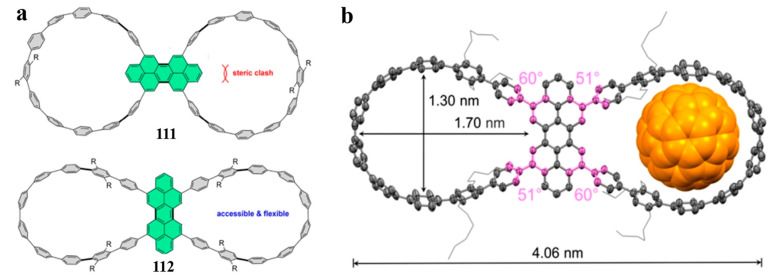
(**a**) The structure of **111** and **112** (**b**) Solid-state structures of a complex of **112** and C_60_ in a 1:1 ratio. Reprinted with permission from ref. [85], copyright 2021 American Chemical Society.

In 2021, Juríček group synthesized two fluorescent bis-CPPs, **111** [84] and **112** [85] (Figure 32a), containing peropyrene cores. The X-ray diffraction (XRD) analysis of single crystals of **111** and **112** demonstrated that they are fully conjugated framework structures with *C*_2_ symmetry. The **111** cannot interact with C_60_ or C_70_ due to spatial resistance. In contrast, **112** can form a 1:1 complex with C_60_ (Figure 32b).

**Figure 33:**
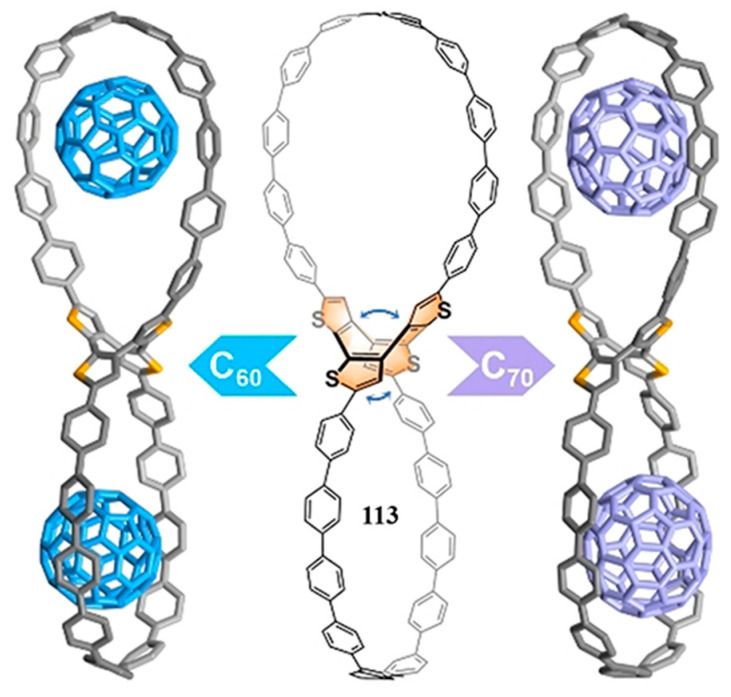
Crystal structure of a complex of **113** formed in a 1:2 ratio with C_60_ or C_70_. Reprinted with permission from ref. [86], copyright 2022 Wiley-VCH.

In 2022, Cong and colleagues synthesized **113** (Figure 33) [86], a fully conjugated bis-CPP containing a flexible cyclooctathiophene core. The crystals of **113** and C_60_ or C_70_ were obtained by slow volatilization of *o*-dichlorobenzene in excess of C_60_ or C_70_, respectively. The assembly structure of **113** with C_60_ or C_70_ in a 1:2 ratio to form a peanut-like topology was isolated and verified.

## 5. Conclusions

In summary, we highlighted recent progress of the bismacrocycle study. The combination of double cavities brings unique properties. To date, bismacrocycles were used in the construction of luminescent materials, supramolecular self-assembly, and supramolecular functional polymers (e.g., gels). The current strategies to generated bismacrocycles included using functionalized linking unit to join two macrocycles (e.g., crown ethers, cyclodextrins, calixarene, cucurbiturates, etc.). In these cases, since both cavities are known, their guest recognition properties are easily accessible for subsequent functionalisation studies. Especially, the simple introduction of functionalized bridging units (e.g., anthracene, azobenzene, dithiophene, etc.) facilitates the property modification of the resulted bismacrocycles and further supramolecular structures. Its simple synthesis steps and low cost are more conducive to pushing the relevant research results to practical applications. Bismacrocycles also can be generated as a whole. This strategy focuses on the introduction of functional structures (e.g., TPE, PDI, etc.) into the bismacrocycles (Table 1). The advantage corresponding to the strategy is some properties (e.g., AIE) can be expected shown in the final product, and mainly used to further supramolecular chemistry exploration.

The design and synthesis of bismacrocycle compounds with outstanding structural novelty and performance is a major challenge in related research. The combination between the art of current bismacrocyclic research and computational methods [87,88,89,90] lead the design and study of specific functionally oriented bismacrocycles. As one of the fast-developing research frontiers, it is expected that bismacrocycle-related chemistry will be further penetrated many fields, including advanced optical materials, disease treatment, and molecular machines, etc. Bismacrocycle study will provide more excellent solutions to achieve the precise construction and properties of complex systems.

## Figures and Tables

**Table 1:** Summary of bismacrocycle shown above.

Bismacrocycle	Sub-Classification	Representative Structure	Applications
Oxabismacrocycle	Bis(crown ether)	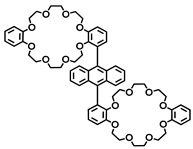	Self-assembly, Supramolecular polymer, Luminescent material, chiral luminescent material.
	Biscyclodextrin	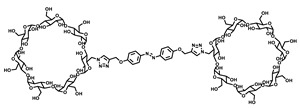	Self-assembly, Supramolecular polymer, Luminescent material, chiral luminescent material, anticancer active materials.
	Biscalixarene	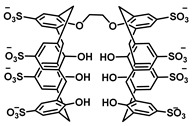	Reversible self-assembly, supramolecular polymer.
	Bispillararene	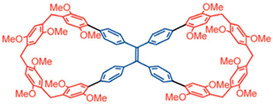	(Stimulus-responsive) luminescent material
	Bishelicarene	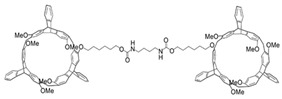	CPL-active supramolecular gels
	Biscucurbituril	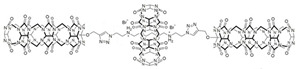	Controlled supramolecular polymer
	Bisheteracalixarenes	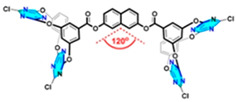	anion-π interaction-directed self-assembly
	Other oxabismacrocycle	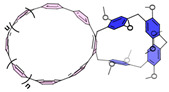	Chiral luminescent material
Azabismacrocycle	Pyridinium bismacrocycle	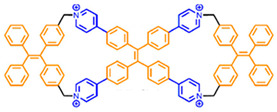	Highly efficient luminescent material, multicomponent assemblies, AIE fluorescent nanomaterial, pollutant detection.
	Biscalix[4]pyrroles	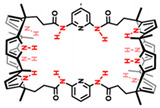	Two/multi-component self-assembly, anion recognition.
	Imidazolium bismacrocycle	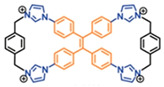	Mechanochromic and photochromic luminescence compounds.
	Azabiscycloparaphenylene	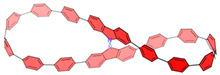	Complex chiral compounds, conformationally interchangeable fluorescent compounds.
Biscycloparaphenylene	Biscycloparaphenylene	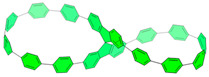	Complex chiral (luminescent) compounds, VOC adsorbent materials, self-assembly,

## Data Availability

Not applicable to this article.

## Sample Availability

Not available.
